# Supplementary material for: Unbiased Quantitative Single-Cell Morphometric Analysis to Identify Microglia Reactivity in Developmental Brain Injury
Source: Life (Basel). 2023 Mar 28;13(4):899. doi: 10.3390/life13040899 (PMC10147015; doi:10.3390/life13040899)
Supplement: Supplementary file 1 [file life-13-00899-s001.zip › life-2211908 -Supplemental figures check.pdf]

A1

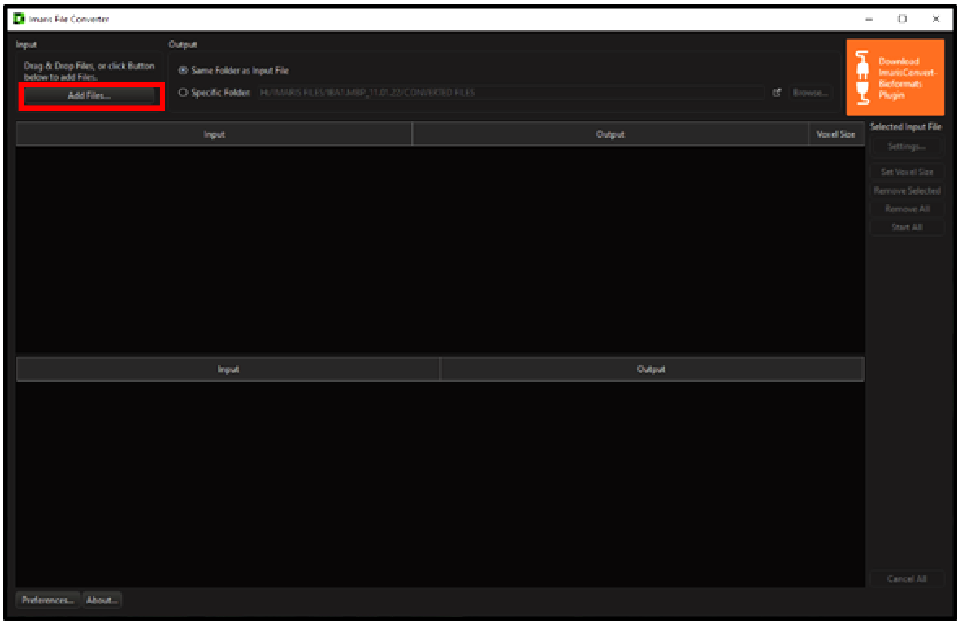

A2

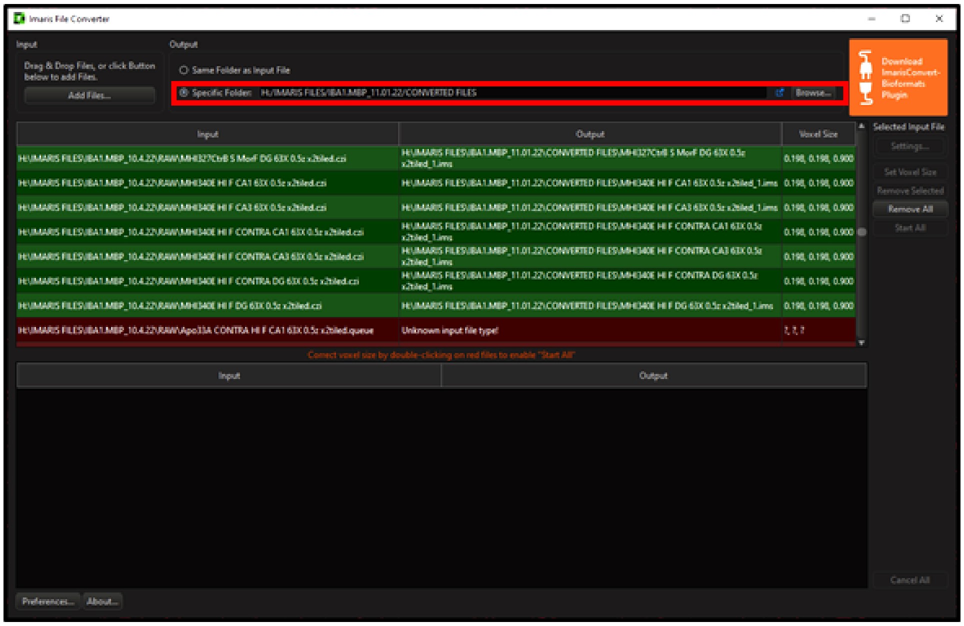

A3

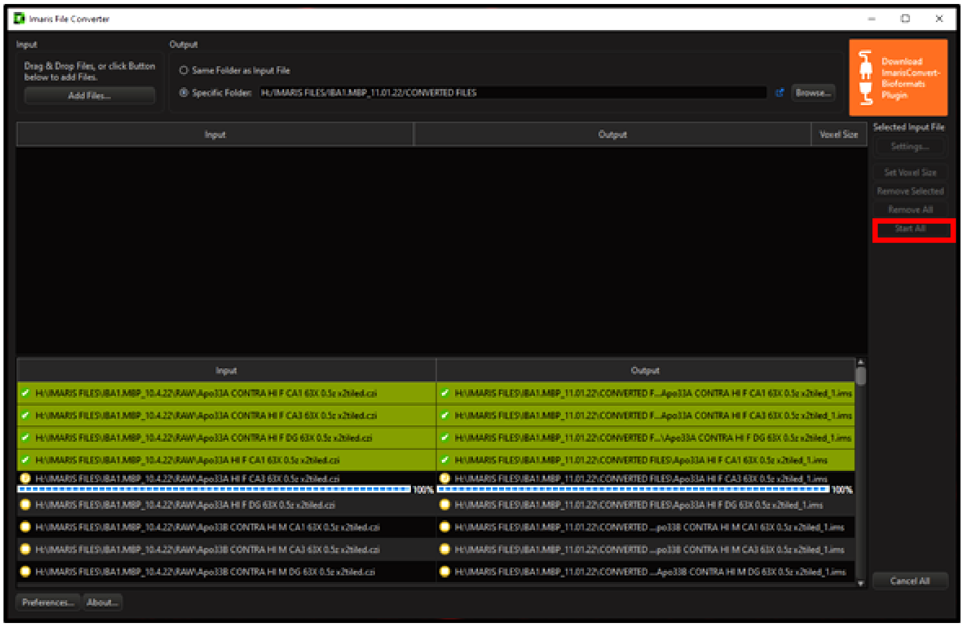

Supplemental Figure S1. Use of Imaris File Converter Software .

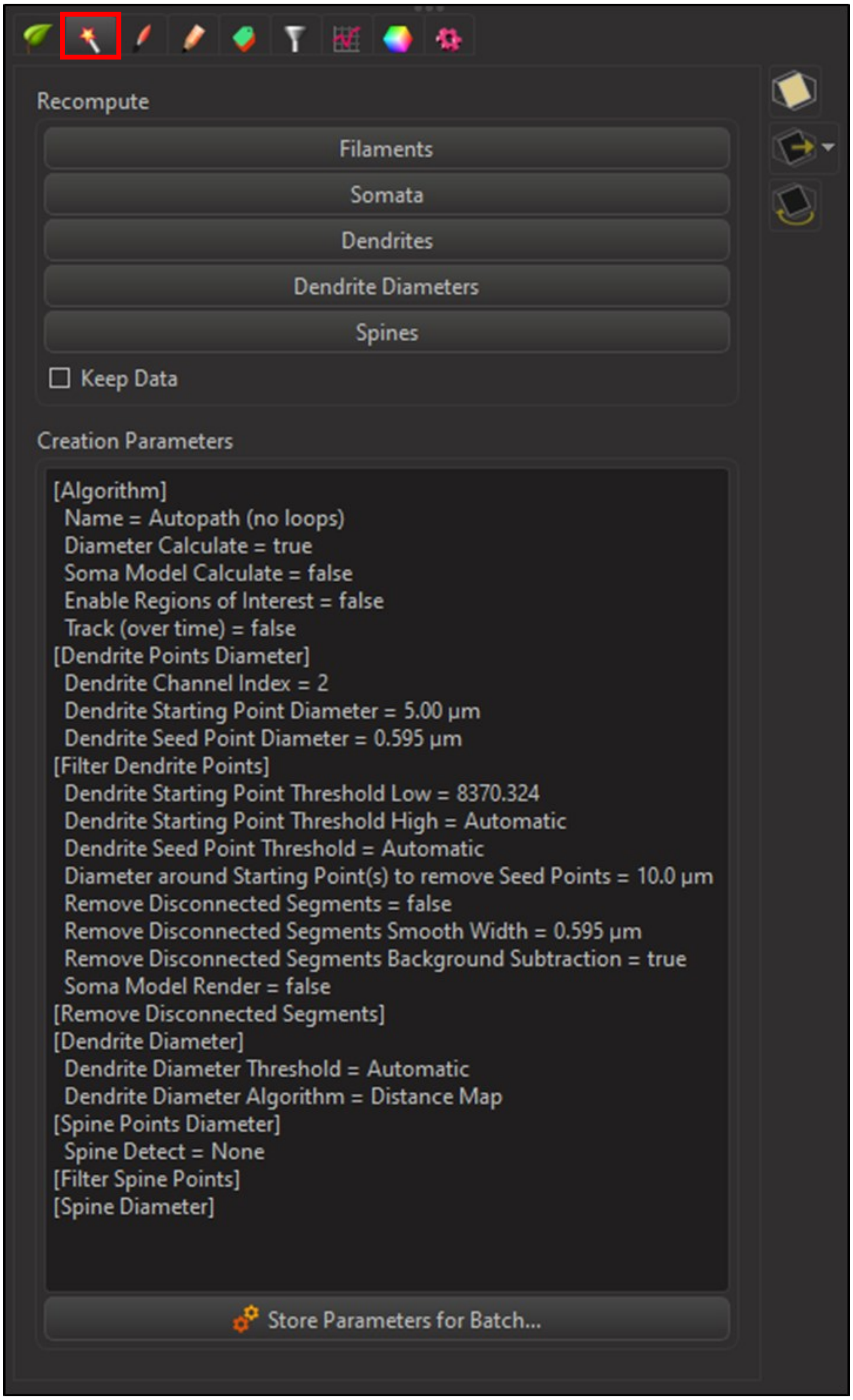

Supplemental Figure S2. After a render is created, create a protocol by saving the object characteristics using the *'Magic Wand'* icon

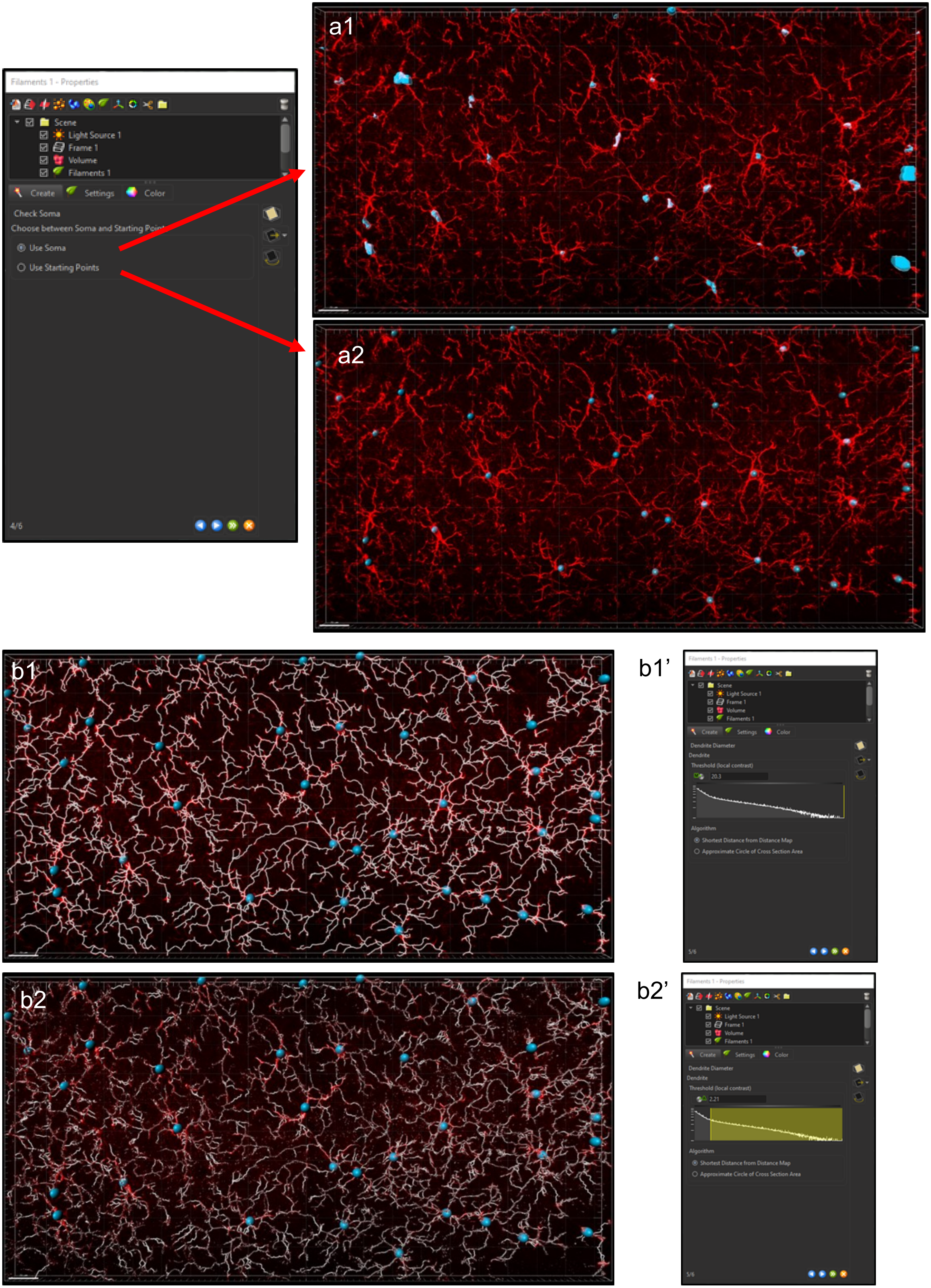

Supplemental Figure S3. Soma determination and setting filament diameter.
